# Supplementary material for: A retrospective two-center cohort study of the bidirectional relationship between depression and tinnitus-related distress
Source: Commun Med (Lond). 2024 Nov 21;4:242. doi: 10.1038/s43856-024-00678-6 (PMC11582723; doi:10.1038/s43856-024-00678-6)
Supplement: Supplementary file 2 — Supplementary Information [file 43856_2024_678_MOESM2_ESM.pdf]

Supplementary Information

A retrospective two-center cohort study of the bidirectional relationship between depression and tinnitus-related distress

Cosima F. Lukas[1], Birgit Mazurek[2], Petra Brueggemann[2], Markus Junghöfer[3], Orlando Guntinas-Lichius[1], Christian Dobel[1,4*]

[1] Department of Otorhinolaryngology, Jena University Hospital, Friedrich-Schiller-University Jena, Jena, Germany
[2] Tinnitus Center, Charité Universitätsmedizin Berlin, Berlin, Germany
[3] Institute of Biomagnetism and Biosignalanalysis, University of Münster, Münster, Germany
[4] Department of Social and Behavioral Sciences, City University of Hong Kong, Hong Kong, China

*Corresponding Author

Table of contents:

SUPPLEMENTARY TABLES .... - 1 -
TABLE 1: COEFFICIENTS AFTER PENALIZATION (ANALYSIS 1) .... - 1 -
TABLE 2: STATISTICAL PARAMETERS - UNCONDITIONAL LCM (ANALYSIS 2) .... - 1 -
TABLE 3: STATISTICAL PARAMETERS - DEPRESSION SEVERITY LCM (ANALYSIS 2) .... - 1 -
TABLE 4: STATISTICAL PARAMETERS - DEMOGRAPHIC LCM (ANALYSIS 2) .... - 2 -
SUPPLEMENTARY RESULTS (ANALYSIS 2) .... - 2 -
UNCONDITIONAL LCM .... - 2 -
DEPRESSION SEVERITY LCM .... - 2 -
DEMOGRAPHIC LCM (TIME-INVARIANT COVARIATES) .... - 2 -
MENTAL-HEALTH LCM (TIME-VARYING COVARIATES) .... - 3 -

Supplementary Tables

Table 1: Coefficients after Penalization (Analysis 1)

Predictors	Coefficients after penalization
age	1.643
sex	-1.534
Tinnitus duration	.
PHQ (somatization)	1.424
PHQ (Depressiveness)	0.290
PHQ (Anxiety)	.
PHQ (Stress)	0.190
TQ (t1)	8.538

Table 2: Statistical Parameters - Unconditional LCM (Analysis 2)

	Est.std	pvalue	ci.lower	ci.upper
**Cor(i_t, s_t)**	-0.264	0.000	-0.321	-0.207
**Cor(i_t, i_D)**	0.640	0.000	0.604	0.677
**Cor(i_t, s_D)**	-0.117	0.000	-0.177	-0.056
**Cor(s_t, i_D)**	-0.093	0.003	-0.154	-0.032
**Cor(s_t, s_D)**	0.483	0.000	0.435	0.530
**Cor(i_D; s_D)**	-0.275	0.000	-0.332	-0.218
**s_t ~1**	-0.081	0.010	-0.143	-0.020
**s_D ~1**	-0.027	0.382	-0.089	0.034

Table 3: Statistical Parameters - Depression severity LCM (Analysis 2)

	Est.std	pvalue	ci.lower	ci.upper
**s_t ~ i_t**	-0.346	0.000	-0.421	-0.272
**s_t ~ i_D**	0.129	0.001	0.052	0.205
**s_D ~ i_D**	-0.339	0.000	-0.413	-0.265

**s_D ~ i_t**	0.100	0.010	0.024	0.177
**s_t ~ s_D**	0.519	0.000	0.474	0.564
**Var(s_t)**	0.921	0.000	0.889	0.952
**Var(s_D)**	0.919	0.000	0.886	0.951
**Cor(s_t, s_D)**	0.519	0.000	0.474	0.564
**Cor(i_t, i_D)**	0.640	0.000	0.604	0.677

Table 4: Statistical Parameters - Demographic LCM (Analysis 2)

	**Est.std**	**pvalue**	**ci.lower**	**ci.upper**
**i_t ~ age**	0.119	0.000	0.059	0.179
**i_t ~ sex**	0.057	0.066	-0.004	0.118
**s_t ~ age**	0.085	0.005	0.025	0.145
**s_t ~ sex**	-0.025	0.405	-0.084	0.034
**s_t ~ i_t**	-0.371	0.000	-0.446	-0.296
**s_t ~ i_D**	0.152	0.000	0.075	0.230
**i_D ~ age**	-0.068	0.029	-0.129	-0.007
**i_D ~ sex**	0.094	0.002	0.033	0.154
**s_D ~ age**	-0.015	0.636	-0.075	0.046
**s_D ~ sex**	-0.055	0.068	-0.114	0.004
**s_D ~ i_D**	-0.337	0.000	-0.413	-0.261
**s_D ~ i_t**	0.102	0.011	0.024	0.180
**Cor(s_t, s_D)**	0.522	0.000	0.477	0.567
**Cor(i_t, i_D)**	0.653	0.000	0.618	0.688

Supplementary Results (Analysis 2)

Unconditional LCM

The average treatment slope factor in tinnitus distress was significantly negative (p = 0·01), whereas the mean treatment slope of depression severity was non-significant (p = 0·382), indicating that the treatment effectively lowers tinnitus distress without significantly improving depression severity. The initial severity levels, as well as the rates of change in tinnitus and depression, are correlated (p = 0·000), indicating that both baseline severity levels and change rates in tinnitus distress and depression are closely associated. Fit estimates cannot be interpreted meaningfully as the model is just identified with df = 0.

Depression severity LCM

Apart from a close correlational relationship between tinnitus distress and depression severity, this model reveals that the intercept–slope effect of initial tinnitus distress on the rate of change in depression severity (p = 0·01) and the intercept–slope effect of the initial depression severity on change in tinnitus severity (p = 0·001) are both significant with positive estimates. This evidence suggests that increased initial symptom severity in one construct slightly mitigates improvement on the other variable. The different significance levels might indicate a higher probability of the effect of depressive symptoms on tinnitus distress. The negative association between initial tinnitus distress and change through therapy, as well as the association between initial depression severity and its respective rate of change, reinforces the finding that increased initial symptom severity is related to enhanced within–symptom improvement (p = 0·000). Fit estimates cannot be interpreted meaningfully as the model is just identified with df = 0.

Demographic LCM (time-invariant covariates)

There was a significant predictive effect of age on the intercept and the slope in tinnitus distress as well as on initial depressiveness. Older age might thus be associated with higher levels of initial tinnitus distress (p = 0·000). The increase in the treatment slope factor due to an increase in age (p < 0·01) suggests that older patients might improve less through therapy.
There were no significant associations between biological sex and the initial level of tinnitus distress (p = 0·066) or its change due to treatment (p = 0·405). The latent intercept and slope factors of tinnitus distress and depression severity stay highly correlated (p = 0·000) when sociodemographic covariates are added to the model. Within-symptom effects also remain significant, as described in the previous model (p = 0·000). The lagged between–symptom treatment effects also stay meaningful with a slightly more pronounced difference in significance levels compared to the depression severity LCM ($p_{s\_D \sim i\_t}$ = < 0·05; $p_{s\_t \sim i\_D}$ = 0·000). Fit indices can again not be interpreted as df = 0.

Mental-health LCM (time-varying covariates)

An extensive analysis of the mental health model is provided in the main article.
