# Supplementary material for: A retrospective two-center cohort study of the bidirectional relationship between depression and tinnitus-related distress
Source: Commun Med (Lond). 2024 Nov 21;4:242. doi: 10.1038/s43856-024-00678-6 (PMC11582723; doi:10.1038/s43856-024-00678-6)
Supplement: Supplementary file 3 — Description of Additional Supplementary Files [file 43856_2024_678_MOESM3_ESM.pdf]

Description of Additional Supplementary Files

File name: Supplementary Data 1

File description: The source data for Figure 1
