# Supplementary material for: A retrospective two-center cohort study of the bidirectional relationship between depression and tinnitus-related distress
Source: Commun Med (Lond). 2024 Nov 21;4:242. doi: 10.1038/s43856-024-00678-6 (PMC11582723; doi:10.1038/s43856-024-00678-6)
Supplement: Supplementary file 5 — REPORTING SUMMARY [file 43856_2024_678_MOESM5_ESM.pdf]

nature portfolio

Corresponding author(s): Christian Dobel

Last updated by author(s): Oct 29, 2024

Reporting Summary

Nature Portfolio wishes to improve the reproducibility of the work that we publish. This form provides structure for consistency and transparency in reporting. For further information on Nature Portfolio policies, see our Editorial Policies and the Editorial Policy Checklist.

Statistics

For all statistical analyses, confirm that the following items are present in the figure legend, table legend, main text, or Methods section.

n/a	Confirmed	
☐	☒	The exact sample size (*n*) for each experimental group/condition, given as a discrete number and unit of measurement
☐	☒	A statement on whether measurements were taken from distinct samples or whether the same sample was measured repeatedly
☐	☒	The statistical test(s) used AND whether they are one- or two-sided<br>*Only common tests should be described solely by name; describe more complex techniques in the Methods section.*
☐	☒	A description of all covariates tested
☒	☐	A description of any assumptions or corrections, such as tests of normality and adjustment for multiple comparisons
☐	☒	A full description of the statistical parameters including central tendency (e.g. means) or other basic estimates (e.g. regression coefficient) AND variation (e.g. standard deviation) or associated estimates of uncertainty (e.g. confidence intervals)
☐	☒	For null hypothesis testing, the test statistic (e.g. *F*, *t*, *r*) with confidence intervals, effect sizes, degrees of freedom and *P* value noted<br>*Give P values as exact values whenever suitable.*
☒	☐	For Bayesian analysis, information on the choice of priors and Markov chain Monte Carlo settings
☐	☒	For hierarchical and complex designs, identification of the appropriate level for tests and full reporting of outcomes
☒	☐	Estimates of effect sizes (e.g. Cohen's *d*, Pearson's *r*), indicating how they were calculated

Our web collection on statistics for biologists contains articles on many of the points above.

Software and code

Policy information about availability of computer code

Data collection	No software was used to collect the data in this study.
Data analysis	Statistical analysis software R version 4.1.0 and the packages "glmnet" version 4.1-8 (for fitting the elastic net regularization path) and the latent variable analysis package “lavaan" version 0.6-16 (for fitting latent growth curve models) were used to analyze the data in this study.

For manuscripts utilizing custom algorithms or software that are central to the research but not yet described in published literature, software must be made available to editors and reviewers. We strongly encourage code deposition in a community repository (e.g. GitHub). See the Nature Portfolio guidelines for submitting code & software for further information.

Data

Policy information about availability of data

All manuscripts must include a data availability statement. This statement should provide the following information, where applicable:

- Accession codes, unique identifiers, or web links for publicly available datasets
- A description of any restrictions on data availability
- For clinical datasets or third party data, please ensure that the statement adheres to our policy

The data are not publicly available due to them containing information that could compromise research participant privacy/consent. Anonymized data are available from the corresponding author CD upon reasonable request. Data are located in controlled access data storage at the University Hospital Jena. Source data underlying the numerical results from all calculated models, figures and tables are provided with the paper and its Supplementary Information files.

Research involving human participants, their data, or biological material

Policy information about studies with human participants or human data. See also policy information about sex, gender (identity/presentation), and sexual orientation and race, ethnicity and racism.

Reporting on sex and gender	Participants' sex was determined through self-reporting and was taken into account during the study's design. Analyses were conducted with sex as a covariate, and descriptive data was reported separately for male and female participants. Although sex differences were not related to the main outcome variable, tinnitus distress, it was observed that improvement in depressive symptoms during treatment was enhanced in female patients, indicating differences in emotional regulation. The data includes Dataset 1 (N= 500, male = 257, female = 243) and Dataset 2 (N = 1016, male = 504, female = 512).
Reporting on race, ethnicity, or other socially relevant groupings	We fully recognize the importance of diversity in research and the psychological burden of discrimination. This study's participants were German-speaking and from similar ethnic backgrounds, reflecting the local population near both tinnitus centers. Therefore, we did not collect data on race or ethnicity. As stated in the methods section, ethnic background is neither an inclusion nor an exclusion criterion. Every individual in need of treatment and fulfilling the mentioned criteria was included.
Population characteristics	See above.
Recruitment	The data was collected from patients who were receiving treatment in one of two specialized day clinics. Inclusion criteria for treatment required chronic tinnitus with a minimum persistence of at least 3 months and the experience of clinically relevant tinnitus-related distress. Patients with acute suicidality or severe psychiatric diagnoses that prevented them from participating in group therapy were not included in the treatment. Different inclusion criteria between day clinics resulted in lower severity levels in the second dataset and, as a result, less treatment-related change in the primary outcome scale. However, this difference is beneficial for one objective of the study, as it helps in identifying predictors relevant across different treatment settings and severity levels. Therefore, we based our investigations on a representative group of treatment-seeking tinnitus patients without making any assumptions about the non-treatment-seeking population.
Ethics oversight	This study protocol was reviewed and approved by the ethics committee of the Jena University Hospital, approval number 4366-03/15, and by the Charité Universitätsmedizin Berlin ethics committee, approval number EA1/115/15 in accordance with the recommendations of the ICH harmonized tripartite guideline for Good Clinical Practice, as well as the Declaration of Helsinki. Written informed consent was obtained from patients to participate in the study.

Note that full information on the approval of the study protocol must also be provided in the manuscript.

Field-specific reporting

Please select the one below that is the best fit for your research. If you are not sure, read the appropriate sections before making your selection.

☐ Life sciences ☒ Behavioural & social sciences ☐ Ecological, evolutionary & environmental sciences

For a reference copy of the document with all sections, see nature.com/documents/nr-reporting-summary-flat.pdf

Behavioural & social sciences study design

All studies must disclose on these points even when the disclosure is negative.

Study description	Retrospective two-center cohort study using quantitative data
Research sample	We used existing treatment data from patients with severe chronic subjective tinnitus, treated in two different interdisciplinary day clinics, representative of the treatment-seeking tinnitus population. This population best provides a basis for investigating the research question regarding the interaction between depressiveness and tinnitus-related distress throughout treatment.<br>Dataset 1: Jena University Hospital; N = 500, male = 257, female = 243; mean age = 55.2 years (SD = 11.5), Dataset 2: Charité Universitätsmedizin Berlin; N = 1016, male = 504, female = 512, mean age = 49.3 years (SD = 11.8). Both datasets contain demographic, tinnitus-related, and mental health-related self-report data.
Sampling strategy	In this study, we used retrospective treatment data collected over several years from all patients who participated in treatment using questionnaires applied within the treatment context of each day clinic. Therefore, the sample size was not predetermined but rather depended on availability. The minimum sample size required for structural equation models with four latent variables and 12 manifest variables to detect medium to small effect sizes with a probability level of 0.05 and a desired statistical power of 0.8 is N = 342. For elastic net analyses, there is no minimum recommended sample size to our knowledge. Therefore, we assume that our sample sizes are sufficient.
Data collection	The participants completed all questionnaires by using pen and paper in the waiting rooms of the day clinics. Researchers were unaware of the hypotheses of the present work at the time of data collection and questionnaire scoring.
Timing	Treatment data had been continuously collected between July 2013 and April 2017 (Jena University Hospital) and between January 2011 and October 2015 (Charité Universitätsmedizin Berlin)

Data exclusions	In the Jena sample, no data were excluded from the analyses. In the Berlin sample, 793 patients have been excluded from the analyses due to values missing completely at random.
Non-participation	No participants dropped out or declined to participate after deciding to undergo treatment.
Randomization	Patients were not allocated into experimental groups.

Reporting for specific materials, systems and methods

We require information from authors about some types of materials, experimental systems and methods used in many studies. Here, indicate whether each material, system or method listed is relevant to your study. If you are not sure if a list item applies to your research, read the appropriate section before selecting a response.

Materials & experimental systems

n/a	Involved in the study	
☒	☐	Antibodies
☒	☐	Eukaryotic cell lines
☒	☐	Palaeontology and archaeology
☒	☐	Animals and other organisms
☐	☒	Clinical data
☒	☐	Dual use research of concern
☒	☐	Plants

Methods

n/a	Involved in the study	
☒	☐	ChIP-seq
☒	☐	Flow cytometry
☒	☐	MRI-based neuroimaging

Clinical data

Policy information about clinical studies

All manuscripts should comply with the ICMJE guidelines for publication of clinical research and a completed CONSORT checklist must be included with all submissions.

Clinical trial registration	We used retrospective clinical data, but conducted no clinical trial.
Study protocol	See above.
Data collection	Tinnitus-related distress and mental health-related questionnaires were administered in the waiting rooms of both day clinics at the beginning and end of treatment using self-report questionnaires (pen and paper). Dataset 1 (Jena University Hospital: between July 2013 and April 2017; Dataset 2 (Charité Universitätsmedizin Berlin): between January 2011 and October 2015.
Outcomes	Tinnitus-related distress, as the primary outcome measure, was assessed using the German version of the Tinnitus Questionnaire (TQ; Goebel & Hiller, 1998). The TQ is frequently used as a standard psychometric instrument for assessing the severity of tinnitus annoyance and impairment and has also been recommended to measure outcomes following interventions.<br>Depressiveness, anxiety, somatic symptoms, and perceived stress have been measured using various validated instruments:<br>Depressiveness: Allgemeine Depressionsskala – long form (ADS-L; Hautzinger M., Bailer M., Hofmeister D., & F., 2012), Berliner Stimmungsfragebogen (BSF; Hörhold, Klapp, & Schimmack, 1993), Patient Health Questionnaire (PHQ; Spitzer et al., 1999), ICD-10-Symptom Rating (ISR; Fischer, Schirmer, Tritt, Klapp, & Fliege, 2011)<br>Anxiety: ISR, PHQ<br>Somatic symptoms: Berlin Complaint Inventory (BI; Hörhold et al., 1997), Short Form-8 Health Survey (SF-8; Bullinger, 1995), ISR<br>Perceived stress: German version of the Perceived Stress Questionnaire (PSQ; Fliege et al., 2005; Levenstein et al., 1993), PHQ

Plants

Seed stocks	-
Novel plant genotypes	-
Authentication	-
